# Peptoids with Antibiofilm Activity against the Gram Negative Obligate Anaerobe, *Fusobacterium nucleatum*

**DOI:** 10.3390/molecules26164741

**Published:** 2021-08-05

**Authors:** Jamie Toole, Hannah L. Bolt, John J. Marley, Sheila Patrick, Steven L. Cobb, Fionnuala T. Lundy

**Affiliations:** 1 Wellcome-Wolfson Institute for Experimental Medicine, School of Medicine, Dentistry and Biomedical Sciences, Queen’s University Belfast, 97 Lisburn Road, Belfast BT9 7BL, UK; j.toole@qub.ac.uk (J.T.); S.Patrick@qub.ac.uk (S.P.); 2 Department of Chemistry, Biophysical Sciences Institute, Durham University, South Road, Durham DH1 3LE, UK; hannah.bolt@astrazeneca.com; 3 Department of Oral Surgery, Royal Victoria Hospital, Grosvenor Road, Belfast BT12 6BA, UK; John.Marley@belfasttrust.hscni.net

**Keywords:** antimicrobial, biofilms, cytotoxicity, peptidomimetic, peptoid, targeted therapeutic

## Abstract

Peptoids (oligo *N*-substituted glycines) are peptide analogues, which can be designed to mimic host antimicrobial peptides, with the advantage that they are resistant to proteolytic degradation. Few studies on the antimicrobial efficacy of peptoids have focused on Gram negative anaerobic microbes associated with clinical infections, which are commonly recalcitrant to antibiotic treatment. We therefore studied the cytotoxicity and antibiofilm activity of a family of peptoids against the Gram negative obligate anaerobe *Fusobacterium nucleatum*, which is associated with infections in the oral cavity. Two peptoids, peptoid **4** (NaeNpheNphe)4 and peptoid **9** (NahNspeNspe)_3_ were shown to be efficacious against *F. nucleatum* biofilms at a concentration of 1 μM. At this concentration, peptoids **4** and **9** were not cytotoxic to human erythrocytes or primary human gingival fibroblast cells. Peptoids **4** and **9** therefore have merit as future therapeutics for the treatment of oral infections.

## 1. Introduction

Currently, a plethora of different management strategies exists for the treatment of Gram negative anaerobic infections in the oral cavity, including the administration of systemic antibiotics. With the global rise in antibiotic resistance, there has been a drive to find alternatives to antibiotics. In recent years, short cationic host defence peptides have been investigated as alternatives to antibiotics, having both antimicrobial and immunomodulatory functions [1,2,3]. Although viewed as nature’s antibiotics, these peptides generally target bacterial cell membranes [1] rather than the specific metabolic pathways targeted by conventional antibiotics. Importantly, this mechanism of action has the advantage that bacteria are less likely to develop resistance [1]. However, the therapeutic translation of peptide treatments has been limited by their cost [4], potential toxicity [1] and their susceptibility to proteolytic degradation [1,5,6].

**Figure 1:**
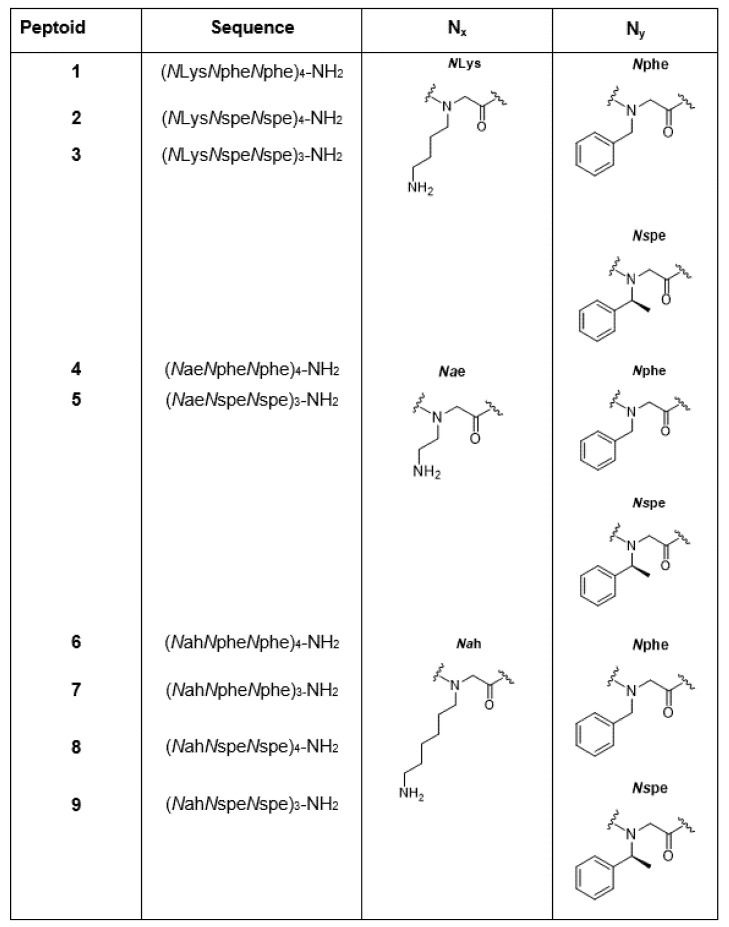
Sequences and pictorial representation of peptoids (**1–9**) analysed in the current study. Peptoid sequences were designed around an NxNyNy subunit, repeated three or four times (9- or 12-residue peptoids, respectively). Within the NxNyNy subunit, Nx is a positively charged lysine-type amine with various side chain lengths; NLys *N*-(4-aminobutyl) glycine, Nae *N*-(2-aminoethyl)glycine) or (Nah *N*-(6-aminohexyl) glycine, whereas Ny is either the chiral aromatic building block; Nspe *N*-(*S*-phenylethyl)glycine or the achiral Nphe N-benzylglycine.

The design and synthesis of oligo-*N*-substituted glycines to generate peptidomimetics termed peptoids [7,8,9] is one potential solution to proteolytic instability, involving modification of the peptide backbone. Whereas peptides have their side chain (R), attached to the *α*-carbon atom, the R group in peptoids is attached to the amide nitrogen, thus rendering peptoids stable to proteolytic degradation [10]. Peptoids have been shown to be very effective antimicrobials against a variety of different microbes [10,11,12,13,14,15,16,17,18,19]. Several families of antimicrobial peptoids have been developed, including those based on repeating subunits [10,20]. One family of amphipathic peptoids, previously studied by our research group [10], was based around an NxNyNy subunit, repeated two, three or four times. Within each subunit, Nx was a positively charged lysine-type amine with various side chains (Nah = *N*-(6-aminohexyl)glycine, NLys = *N*-(4-aminobutyl)glycine or Nae = *N*-(2-aminoethyl)glycine), and Ny was either the chiral aromatic monomer Nspe (*N*-(*S*-phenylethyl) glycine) or the achiral Nphe (*N*-Benzylglycine) [10]. The chiral peptoid monomer Nspe has been shown to help stabilise the helical secondary structure of the linear peptoid, which is known to improve anti-microbial activity [15]. Several of the peptoids from this family (peptoids **1**–**9**, Figure 1) have been shown to be effective against *Escherichia coli* biofilms [10], commonly associated with medical device infections.

The antimicrobial efficacy of peptoids has not previously focused on Gram negative obligately anaerobic microorganisms. *Fusobacterium nucelatum* is a Gram negative obligate anaerobe that plays an important role in the developing oral plaque biofilm, particularly in the transition from the commensal Gram positive aerobes found in healthy mouths to the pathological Gram negative anaerobes associated with the pathogenesis of infections such as periodontal disease and peri-implantitis in the oral cavity [21]. With periodontal disease reported to affect 45% of the population [22] and peri-implantitis reported to affect between 1–47% of dental implants [23], new treatments for oral anaerobic infections are currently in demand. In the development of oral biofilms, *F. nucleatum* tends to act as a ‘bridging’ microbe, facilitating the adherence of more pathogenic Gram negative anaerobes (late colonisers) to the commensal microbes (early colonisers). Treatment and eradication of *F. nucleatum* could limit colonisation by more pathogenic microbes and therefore limit development of infection.

Acute, Gram negative anaerobic infections in the oral cavity may require the use of antibiotics, which have been reported to be over-prescribed by dentists [24]. Antibiotic resistance is now emerging in oral pathogens, and it was shown that 16% of *F. nucleatum* strains are resistant to amoxicillin and 36% of *F. nucleatum* strains are resistant to clindamycin [25]. As possible alternatives to the use of antibiotics, our research group and others have focused on peptide-based antimicrobials with efficacy against *F. nucleatum* [26,27,28]. Peptides from the African volcano frog *Xenopus amieti* (Pipidae), were previously shown by us to have antimicrobial activity against *F. nucleatum* [26]. More recently, ultra-short peptide hydrogels [27] and a peptide-loaded poloxamer-based microemulsion gel [28] were also shown to be efficacious against *F. nucleatum*. Moreover, clinical trials have reported efficacy for peptide-based treatments of oral infections [29].

The aim of this research was to determine the cytotoxicity of a series of peptoids against human erythrocytes and human primary cells, and to assess their antibiofilm efficacy against *F. nucleatum*. To our knowledge, this is the first report to study peptoid efficacy against an anaerobic microbe.

## 2. Results

### 2.1. Haemolytic Effect of Peptoids on Human Erythrocytes

**Figure 2:**
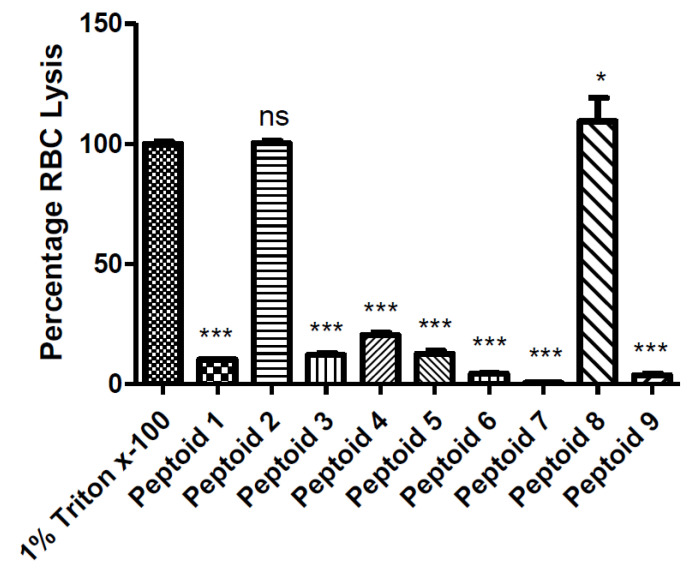
Percentage lysis of human erythrocytes by peptoids **1–9** (100 µM) compared with positive control (1% Triton x-100). (***: *p* < 0.001; *: *p* < 0.5; ns: *p* > 0.05). One-way ANOVA followed by Dunnett’s multiple comparison test. (*n* = 3 independent experiments, 3 replicates in each).

Peptoids **2** and **8** lysed similar (peptoid **2**) or higher (peptoid **8**) percentages of human erythrocytes compared to the Triton x-100 positive control (Figure 2) and were not studied further. The remaining peptoids displayed variable haemolytic activity against human erythrocytes; peptoids **1**, **3**, **4** and **5** lysed 10% or more of erythrocytes; and peptoids **6**, **7** and **9** lysed less than 5% of erythrocytes.

### 2.2. Cytotoxicity of Peptoids against Human Gingival Fibroblasts

**Figure 3:**
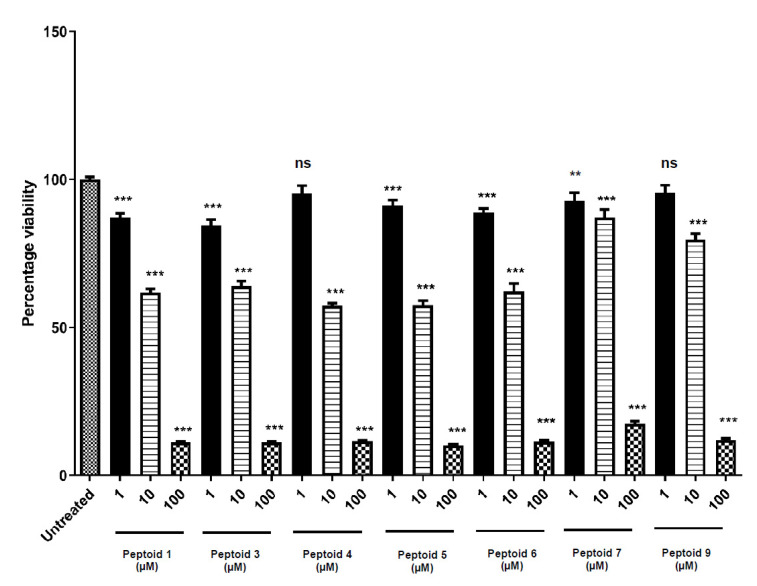
Cell viability as determined by MTT assay following treatment of gingival fibroblasts with peptoids **1**, **3–7** and **9** (1, 10 and 100 µM), (***: *p* < 0.001; **: *p* < 0.01; ns: *p* > 0.05). One-way ANOVA followed by Dunnett’s multiple comparison test. (n = 3 independent experiments, 6 replicates in each).

In addition to cytolysis of human erythrocytes, peptoids were tested for their effects on the viability of human primary gingival fibroblasts [30], since these cells are relevant should peptoids be translated into clinical practice for the treatment of periodontal disease and/or peri-implantitis. Of the seven peptoids tested (**1**, **3**–**7** and **9**) all significantly decreased viability of gingival fibroblasts at concentrations of 10 µM and 100 µM, whereas at a concentration of 1 μM, peptoids **4** and **9** were shown not to alter gingival fibroblast viability compared with untreated controls (Figure 3).

### 2.3. Biofilm Inhibition by Peptoids

**Figure 4:**
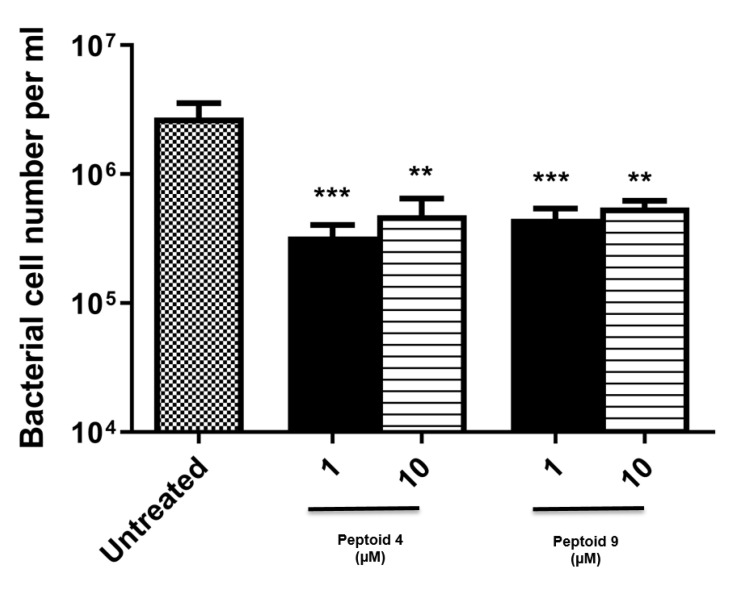
*F. nucleatum* cell numbers in a biofilm inhibition assay, following treatment with peptoids **4** and **9** at 1 µM and 10 µM. Cell numbers were measured by propidium monoazide(PMA)-qPCR for detection of live cells only (***: *p* < 0.0005; **: *p* < 0.05 One-way ANOVA followed by Dunnett’s multiple comparison test, n=3 independent experiments, 3 replicates in each).

Based on the initial cytotoxicity data (Figure 2 and Figure 3) peptoids **4** and **9** were then tested for efficacy in inhibiting *F. nucleatum* biofilm formation in 96 well microtitre plates under anaerobic conditions. Peptoids **4** and **9** showed a statistically significant reduction in biofilm formation at both 1 µM and 10 µM concentrations (Figure 4). Interestingly, there did not appear to be enhanced biofilm inhibition at 10 µM versus 1 µM for both peptoids studied (Figure 4).

## 3. Discussion

The family of peptoids studied herein had previously been examined for their cytotoxicity against HepG2 epithelial and HaCaT keratinocyte cell lines [10], but had not been tested for their cytocoxicity against human erythrocytes or primary cells. The majority of these peptoids (15/18 peptoids tested) previously displayed no toxicity to either the HepG2 epithelial or HaCaT keratinocyte cell lines at a concentration of 100 µM [10]. Interestingly, however, peptoids **2** and **8** were found to be toxic to both cell lines at concentrations between 20–40 µM [10]. In agreement with this finding, we report in the current study that peptoids **2** and **8** had the highest levels of human erythrocyte haemolysis.

The remaining peptoids **1**, **3**–**7** and **9** were then assessed for their potential effects on gingival fibroblast viability. These cells have an important role for healing following infection or surgical incision in the oral cavity. If healing is delayed, it can increase bacterial seeding onto the implant surface. In oral wounds, fibroblasts start to migrate to the site within 24 h and begin production of granulation tissue during the early phase of wound healing [31]. Fibroblasts then produce new connective tissue as well as differentiating to myo-fibroblasts to allow for wound contraction, the final stage of wound healing, which is generally completed within 4–8 days [31]. Peptoid **4** (NaeNpheNphe)_4_-NH_2_ and peptoid **9** (NahNspeNspe)_3_-NH_2_ were the only members of the peptoid family that did not significantly reduce cell viability of human gingival fibroblasts at a concentration of 1 µM. Immortalised cell lines have often been used to test the cytotoxicity of potential therapeutics, including peptoids [10,20], whereas primary cell cultures were used in the current work. It is well recognised that immortalised cell lines are more resistant to cytotoxic chemicals than cultured primary cells [32]. Both HepG2 and HaCaT have been reported to be significantly less sensitive to many toxins [33,34]. This may be the result of the genetic modifications, and resultant changes in characteristics that enable immortalised cell lines to be continuously sub-cultured [34]. As a result, it is suggested that immortalised cell lines are a poor substitute for relevant primary cells in cytotoxicity studies. While primary cell cultures are short-lived when compared with immortalised cell lines, they are closer characteristically to native fibroblasts and thus provide a more sensitive in vitro model for cytotoxicity studies. Our data, employing human primary gingival fibrobalst cultures, clearly demonstrate this increased sensitivity and underline the importance of primary cell cultures in cytotoxicity assays.

Peptoids **4** and **9** showed a statistically significant reduction in *F. nucleatum* biofilm formation as determined by propidium monoazide (PMA)-qPCR. The technique of PMA-qPCR allows quantification of the number of living cells within biofilms. PMA binds covalently to double-stranded DNA that is not protected by the cell membrane in viable microbes. DNA bound to PMA will not be quantified by qPCR, and thus the PMA-qPCR method will quantify viable cells only. PMA-qPCR has advantages over conventional colony forming unit (CFU) counting techniques because CFU quantification of microbes within biofilms can significantly underestimate the total live cell count, due to the presence of ‘viable but non-culturable’ (VBNC) microbes. The VBNC bacterial cells are metabolically active but do not grow and divide in routine laboratory culture media. It is known that many species of microbes have the capacity to enter the VBNC state, depending on external environmental conditions [35], including antimicrobial treatment regimens [36]. Thus, antimicrobial efficacy could be over-estimated by traditional CFU assays if VBNC microbes are present after treatment, because by definition these cells will not form CFUs for enumeration. The log_10_ reductions in biofilms observed following peptoid treatment therefore represent significantly decreased cell numbers, which if translated in vivo could allow the host immune system to act more efficiently against the remaining biofilm.

In conclusion, we report the identification of two peptoids with efficacy against *F. nucleatum* biofilm formation in vitro. Effective antimicrobial therapy against anaerobes is particularly important in view of their importance as human pathogens [37], and peptoid treatment may represent a novel way to reduce disease-causing biofilms.

## 4. Materials and Methods

### 4.1. Peptoid Preparation

Peptoids **1**–**9** (Figure 1), which were previously prepared as described in detail by Luo et al. [10], were initially screened for their potential cytotoxicity against human erythrocytes at a working concentration of 100 µM. All peptoids were soluble in aqueous solutions. Peptoid stock solutions were prepared in distilled deionized water and subsequent diluted in phosphate-buffered saline (PBS) to obtain working concentrations for use in cytotoxicity and biofilm assays.

### 4.2. Haemolytic Assay

The haemolytic assay was employed to determine the potential cytotoxicity of the peptoids against human red blood cells. Blood samples were collected with ethical approval and informed consent in vacutainer containers with dipotassium ethylene- diaminetetraacetic acid (K2-EDTA). Blood was then transferred to a 50 mL tube and centrifuged at 1200× *g* for 5 min at room temperature. The supernatant was removed and PBS was added to the original volume of sample obtained. This step was repeated a further 3 times. The red blood cells were then diluted to 8% of their original volume for use in the haemolytic assay. A 1% solution of Triton x-100 in PBS served as a positive control, and PBS was used as a negative control.

Peptoids **1**, **2**, **3**, **4**, **5**, **6**, **7**, **8** and **9** were diluted in PBS to 200 µM. A total volume of 100 µL of each peptoid was placed in the wells of a v-bottomed 96-well microtitre plate. Red blood cells (100 µL), prepared as outlined above, were then added. The final peptoid concentration in each well was therefore 100 µM. The plate was sealed with an adhesive film to prevent evaporation and incubated at 37 °C for 1 h. Following this time, the plate was centrifuged at 4000 RPM for 5 min to pellet unlysed red blood cells. Following this step, 100 µL of supernatant was carefully removed from each well and added to a flat-bottomed 96-well microtitre plate. The absorbance was read at 450 nm using a Tecan Genios (Männedorf, Switzerland) plate reader, and the percentage of red blood cells lysed was calculated.

### 4.3. 3-(4,5-Dimethylthiazol-2-yl-2,5-diphenyltetrazolium Bromide (MTT Assay)

Cultured primary gingival fibroblast cells were obtained with ethical approval and informed consent, and were grown as previously described [30]. Cells were seeded in 96-well plates at a concentration of 1 × 10^5^ cells/mL (100 µL per well). Plates were incubated at 37 °C for 48 h to allow cells to reach confluence. Peptoids **1**, **3**, **4**, **5**, **6**, **7** and **9** were diluted to 200 µM, 20 µM and 2 µM. The culture medium was removed and replaced with 50 µL of Dulbecco’s Modified Eagle’s Medium (DMEM) and 50 µL of the relevant peptoid. The cells were incubated for 24 h at 37 °C. After this time, 10 µL of 3-(4, 5- dimethylthiazol-2-yl)-2,5-diphenyltetrazolium bromide (MTT) reagent (Sigma-Aldrich, Poole, UK) was added to each well and the plate was incubated for a further 3 h at 37 °C. The medium was removed by aspiration and the wells were allowed to dry for 20 min. Then, 200 µL of dimethyl sulfoxide (DMSO) (Sigma-Aldrich, Poole, UK) was added to all wells and the plate was placed in an incubator for 30 min. The absorbance of the wells was read in a Tecan Genios (Männedorf, Switzerland) plate reader at 510 nm after 60 s of orbital shaking.

### 4.4. Biofim Inhibition Assay

The *F. nucleatum* type strain (NCTC 10562, originally isolated from human, inflamed gingival tissue) was obtained from the National Collection of Type Cultures. The optical density (OD) of an overnight culture was measured spectrophotometrically at 570 nm and diluted with fresh broth to an OD of 0.30–0.35. The culture was further diluted 1 in 50 with anaerobic broth, giving a final inoculum concentration of approximately 5 × 106 CFU/mL. A biofilm inhibition assay was undertaken in a 96-well plate format. Peptoids **4** and **9** were added along with the inoculum (90 µL of inoculum in broth and 10 µL of peptoid solution) to each well, to yield peptoid concentrations of 1 µM and 10 µM. A solution containing 10 µL of PBS and 90 µL of anaerobic broth acted as an untreated control. Biofilms were allowed to develop in the presence of peptoids **4** or **9** for 48 h. After 48 h biofilm development, planktonic cells were removed by gently washing three times with PBS and then replacing the broth with 100 µL of fresh anaerobic broth prior to a further 48 h incubation for biofilm maturation. After this time, the wells were gently washed three times with 100 µL of PBS to remove planktonic cells. Plates were then sealed with parafilm, and the biofilms were dislodged from the wells in an ultrasonic bath for 20 min. Plates were then returned to the anaerobic cabinet and the dislodged biofilm samples were collected in Eppendorf tubes.

### 4.5. Propidium Monoazide (PMA–Modified) qPCR

Propidium monoazide (PMA)-qPCR was undertaken essentially as previously described [38]. Briefly, PMA (Biotium, Fremont, CA, USA), was diluted 1 in 50 with PBS. The diluted PMA was then added to the dislodged biofilm samples in a 1:1 ratio in an Eppendorf tube, to give a final PMA concentration of 200 µM. The Eppendorfs were incubated at 37 °C for 10 min in the dark and then placed under a broad spectrum LED floodlight (ELRD, HL60 ^0 LEDx Max 0.1V) for 10 min. During this time, the Eppendorfs were inverted several times. Bacterial DNA was then extracted using the Qiagen blood and tissue kit (Germantown, MD, USA) according to the manufacturer’s instructions. Briefly, bacterial samples released from biofilms were centrifuged at 14000 RPM for 6 min, and the supernatant was removed. Bacterial pellets were resuspended in 200 µL of AL buffer, and 25 µL of proteinase K was added to each Eppendorf. The suspensions were then heated to 56 °C for 30 min with shaking. Following this, 200 µL of ethanol was added to each Eppendorf and mixed gently. The contents were placed in fresh spin columns in 2 mL collection tubes. The spin columns were centrifuged at 8000 RPM for 2 min. The flow-through and the collection tubes were discarded. The spin columns were placed in fresh collection tubes, and 500 µL of AW1 buffer was added to each column. The spin columns were centrifuged at 8000 RPM for 2 min. Again, the flow-through and the collection tubes were discarded. The spin columns were placed in fresh collection tubes, and 500 µL of AW2 buffer was added. The spin columns were centrifuged at 16000 RPM for 3 min. The flow-through and the collection tubes were discarded. The spin columns were placed in fresh collection tubes, and 100 µL of AE buffer was added to each. These were incubated at room temperature for 1 min. The tubes were then centrifuged at 8000 RPM for 1 min. The extracted DNA samples were then transferred to DNase-free Eppendorfs. Finally, qPCR was undertaken using *F. nucleatum* specific primers (Table 1), using the reaction formulations detailed in Table 2 and the reaction conditions outlined in Table 3.

## Figures and Tables

**Table 1:** Primer sequences for *F. nucleatum*.

	Sequence
*F. nucleatum* Forward Primer	CAACCATTACTTTAACTCTACCATGTTCA
*F. nucleatum* Reverse Primer	GTTGACTTTACAGAAGGAGATTATGTAAAAATC

**Table 2:** qPCR master mix formulation.

	Volume Per Reaction	Final Concentration
Lightcycler 480 SYBR Green I Master (x2)	5 µL	X1
Forward Primer (10 µM)	0.1 µL	100 nM
Reverse Primer (10 µM)	0.1 µL	100 nM
Nuclease free water	3.6 µL	
DNA template	1.2 µL	
**Total Reaction Volume**	**10 µL**	

**Table 3:** qPCR reaction conditions for *F. nucleatum*.

Cycles	Temperature	Hold Time
1	95 °C	5 min
65	95 °C	10 s
65	55 °C	10 s
65	72 °C	10 s
*Melt analysis*		
1	95 °C	5 s
1	96 °C	1 min
1	97 °C	1 min

## Data Availability

The data that support the findings of this study are available from the corresponding authors upon reasonable request.

## Sample Availability

Samples of the compounds are available from the corresponding authors upon reasonable request.
